# Cloning and Functional Analysis of *BcMYB101* Gene Involved in Leaf Development in Pak Choi (*Brassica rapa* ssp. *Chinensis*)

**DOI:** 10.3390/ijms21082750

**Published:** 2020-04-15

**Authors:** Hualan Hou, Changwei Zhang, Xilin Hou

**Affiliations:** 1 State Key Laboratory of Crop Genetics & Germplasm Enhancement, Key Laboratory of Biology and Genetic Improvement of Horticultural Crops in East China, Ministry of Agriculture, Department of Horticulture, Nanjing Agricultural University, Nanjing 210095, China; 2017204021@njau.edu.cn (H.H.); changweizh@njau.edu.cn (C.Z.); 2 Engineering Research Center of Germplasm Enhancement and Utilization of Horticultural Crops, Nanjing Agricultural University, Nanjing 210095, China

**Keywords:** GAMYB, leaf development, pak-choi, *BcMYB101*, GA, trans-activation activity

## Abstract

As one of the largest transcription factor families, MYB transcription factors are widely present, and they are involved in a diverse range of physiological activities in plants, such as leaf development. GAMYB genes belong to the R2R3-MYB subfamily, which includes the *MYB33*/*65*/*101* gene, and these genes are studied well in seed germination and flowering, but their roles in leaf development are poorly understood. In the current study, we isolated a GAMYB transcription factor from pak choi, *BcMYB101*, and analyzed its characteristics and function. The sequence structure analysis indicated that *BcMYB101* has a highly conserved R2R3 DNA-binding domain in the N-terminal region and three GAMYB-specific motifs (Box1, Box2, and Box3). The expression pattern of diverse tissues revealed that *BcMYB101* has a higher transcript level in the petiole, leaf, root, and floral organs. Furthermore, the expression level was significantly elevated after GA (gibberellin) treatment, suggesting that the *BcMYB101* response was positively regulated by GA. Subcellular localization exhibited that *BcMYB101* was only present in the nuclear region, consistent with the characterization of the transcription factor. The overexpression of *BcMYB101* elucidated that *BcMYB101* increased leaf number and resulted in downward-curling cauline leaves. Moreover, the virus-induced *BcMYB101* silencing displayed that *BcMYB101* is involved in the regulation of curly leaves. Furthermore, we discovered that *BcMYB101* has two trans-activation activities and one interaction protein, *BcTCH4*, using a trans-activation activity assay and a yeast two-hybrid assay, respectively. In this study, we firstly isolated the *BcMYB101* gene and explored its function in leaf development, thereby providing a solid foundation for further research on the regulatory mechanism of leaf shape in *Brassica* or other species.

## 1. Introduction

Transcription factors mediate plant growth and development, as well as physiological metabolism, to adapt to changes in the external environment via regulating the expression of target genes at the transcriptional level. Transcription factors can be divided into disparate families based on the structure of the DNA-binding domain, such as the MYB protein, high-mobility group (HMG) protein, basic leucine zipper (bZIP) protein [1], heat-shock protein (HSP), zinc-finger protein [2], MADS-box protein [3], AP2 (APETALA2)/EREBP (ethylene-responsive element binding protein) protein [4], basic helix–loop–helix (bHLH) protein [5], homeodomain protein [6], etc. The MYB superfamily, as one of the largest transcription factor families in plants, plays an important role in diverse biological functions, including the plant hormone and environmental factor response, the coloration of flesh and pericarp [7], cell cycle and cell differentiation regulation [8], anthocyanin formation and secondary metabolism [9], and the morphogenesis of organs such as leaves [4]. The v2MYB gene was the first discovered MYB transcription factor from the leukemia viruses AMV and E26 in bird, and the first plant MYB gene identified was *ZmMYBC1* from *Zea mays* [10]. Interestingly, there are more MYB transcription factors in plants than in animals, and hundreds of MYB transcripts were identified in plants to date. MYB transcripts are a hot topic in the field of botany research, and the research progress is rapid due to the numerous members and diverse functions.

MYB TFs (transcription factors) can be categorized into four subclades depending on the number of MYB domains in *Arabidopsis thaliana* [11]: (1) 1R-MYB (R1/2, R3), (2) R2R3-MYB, (3) 3R-MYB (R1R2R3-MYB), and (4) 4R-MYB. Among them, R2R3-MYB has the largest number of members. So far, 126 R2R3-MYB members were identified among 339 MYB TFs in *Arabidopsis thaliana*, and about 109 R2R3-MYB members were identified among 230 MYB TFs in rice [12]. In addition, more than 200 and 80 MYB transcription factors were found in cotton and maize, respectively. Furthermore, the R2R3-MYB proteins are classified into 22 subgroups based on sequence conservation within the C-terminal region, and most of them have a transcriptional activation domain [13]. GAMYB belongs to the 18th subgroup of the R2R3-MYB family, consisting of *MYB33*, *MYB65*, *MYB81*, *MYB97*, *MYB101*, *MYB104*, and *MYB120* [11,14]. GAMYB, as a downstream gene of the DELLA protein (a subfamily of the GRAS family), was initially isolated from the aleurone layer in barley, which responds to GA signal transduction [15]. Subsequently, it was isolated and identified in *Arabidopsis*, rice, oats, Asian cotton, strawberry, tomato, cucumber, and corn [16,17,18,19,20,21,22]. The GAMYB proteins not only share a common R2R3 conserved DNA-binding domain, but they also have unique motifs termed Box1, Box2, and Box3. The GAMYB protein directly binds to specific sequences (binding region) in the promoter of GA-responsive genes and trans-activates them [15]. GAMYB plays an essential role in flower induction, and the expression of GAMYB and *LEAFY* (*LFY*) increased with exogenous GA_4_ treatment under short-day conditions in *Arabidopsis thaliana* [23]. Moreover, GAMYB plays a major role in regulating the development of floral organs, thereby affecting the growth of stamens, anthers, and pollen in plants [24,25]. In *Arabidopsis*, GAMYB-like genes *MYB33*/*65*/*101* mediate GA signaling in petiole elongation and flowering responses [23]. Three out of seven GAMYB members (*MYB97*, *MYB101*, and *MYB120*) act as male factors involved in pollen tube reception, possibly by regulating the transcription of downstream genes [26]. GAMYB-like genes (*MYB33*/*65*) were regulated by *miR159* to promote the progression of programmed cell death and to inhibit growth in the aleurone [27]. Additionally, *MYB33* and *MYB101* transcription factors play a role in improving hypersensitivity to ABA (abscisic acid) and soil drought tolerance in *Arabidopsis* and tomato [28]. In rice, loss-of-function mutants of OsGAMYB led to the abnormal development of the stamen and anther [24,25]. The OsGAMYB protein is also required for the generation of α-amylase in aleurone layer cells in rice [24].

*Brassica* and *Arabidopsis* have a close relationship, with both belonging to the *Brassicaceae* family. As a member of the genus *Brassica*, *B. rapa* subgenomes underwent evolution through genome fractionation from *Arabidopsis* [29]. Pak-choi (*Brassica rapa* ssp. *Chinensis*), belonging to the *B. rapa* family, is a major leafy vegetable and is widely cultivated in Asia [30]. Leaf morphology is an important agronomic trait with a great impact on pak choi architecture and productivity. *MYB101*, as a member of the GAMYB-like gene family, together with *MYB33*/*65*, regulates the expression of genes that are induced by GA during seed germination [31]. Meanwhile, *MYB101*/*ABS7* also plays a major role in leaf lamina development in *Arabidopsis* [32]. Despite the functions of GAMYB genes being reported in several species, they are yet to be fully ascertained in pak choi. 

Here, we studied a *GAMYB-*like gene from pak choi, *BcMYB101*, the protein of which is targeted in the nucleus. We show that *BcMYB101* has a highly conserved R2R3 domain and GAMYB-specific motifs (Box1, Box2, and Box3). The spatio-temporal expression profile of *BcMYB101* manifested that *BcMYB101* was expressed in various tissues, especially in the floral organs, which is consistent with its widely studied function in flower induction and development. Furthermore, the transcript levels of *BcMYB101* were gradually increased with the application of GA3, indicating that *BcMYB101* can be induced and positively regulated by GA. To investigate the influence of *BcMYB101*, we performed overexpression and silencing assays of *BcMYB101* in *Arabidopsis* and pak choi, respectively. The results indicated that *BcMYB101* possibly participates in the development of curly leaves in pak choi. To further explore the regulatory network of the *BcMYB101* protein, we used yeast two-hybrid assays to identify its trans-activation domain and to screen its possible interaction proteins; one candidate protein, *BcTCH4*, was found to interact with *BcMYB101* in yeast. These findings extend our understanding of *MYB101* in Cruciferae plants, suggesting that *BcMYB101* acts as a nuclear-target protein, involved in gibberellin signaling and leaf development in pak choi.

## 2. Results

### 2.1. Identification of BcMYB101 and Sequence Alignment

The *BcMYB101* cDNA was isolated from pak choi cultivar “*suzhouqing*”, encoding a protein of 479 amino acids with a predicted molecular mass of 54.07 kDa and theoretical pI of 5.36. The instability index (II) and grand average of hydropathicity (GRAVY) were computed to be 49.92 and −0.885, respectively, which classified *BcMYB101* as an unstable and hydrophilic protein. 

**Figure 1:**
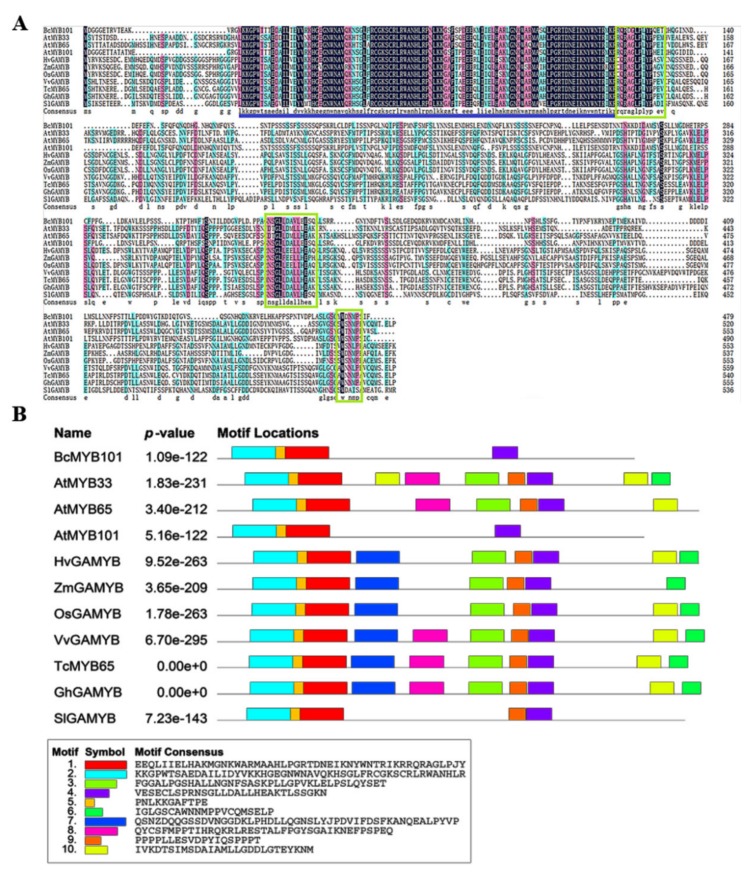
Multiple sequence alignment and motif compositions of GAMYB genes. (**A**) Sequence alignment of GAMYB proteins in *Hordeum vulgare* (*Hv*), *Solanum lycopersicum* (*Sl*), *Oryza sativa* (*Os*), *Vitis vinifera* (*Vv*), *Zea mays* (*Zm*), *Arabidopsis thaliana (At*), *Gossypium hirsutum* (*Gh*), *Theobroma cacao* (*Tc*), and pak choi (*Brassica rapa* ssp. *Chinensis*; *Bc*). The conserved R2R3 domain of GAMYB and GAMYB-like proteins is marked above the blue line, while conserved Box1, Box2, and Box3 are represented in green boxes. (**B**) Distribution of the conserved motifs of GAMYB genes. The sequences of the 10 motifs are exhibited at the bottom.

Multiple sequence alignment of the GAMYB and GAMYB-like proteins from Hordeum vulgare, Solanum lycopersicum, Oryza sativa, Zea mays, Vitis vinifera, Gossypium hirsutum, Theobroma cacao, Arabidopsis thaliana, and pak choi showed that the BcMYB101 protein contains a highly conserved R2R3 domain in the N-terminal portion and three GAMYB-specific conserved regions Box1, Box2, and Box3 (Figure 1A). Furthermore, multiple sequence alignment indicated that the BcMYB101 protein shares 24.22%, 24.74%, 24.39%, and 25.26% identity with OsGAMYB from rice, ZmGAMYB from corn, HvGAMYB from barley, and SlGAMYB from tomato, respectively, while it shares more than 60% identity with AtMYB101 from Arabidopsis, thereby further confirming the conservation of Cruciferae plants in evolution. The conserved motif distribution displayed that motif1, motif2, and motif5 in the N-terminal region and motif4 in the C-terminal region were highly conserved in all GAMYB and GAMYB-kike proteins (Figure 1B). Additionally, the secondary structure, predicted using PSIPRED 4.0, exhibited that the BcMYB101 protein mainly comprised alpha helices (115 amino acids, 24%) and random coils (364 amino acids, 76%) (Figure S1). 

### 2.2. Phylogenetic Analysis of GAMYB 

**Figure 2:**
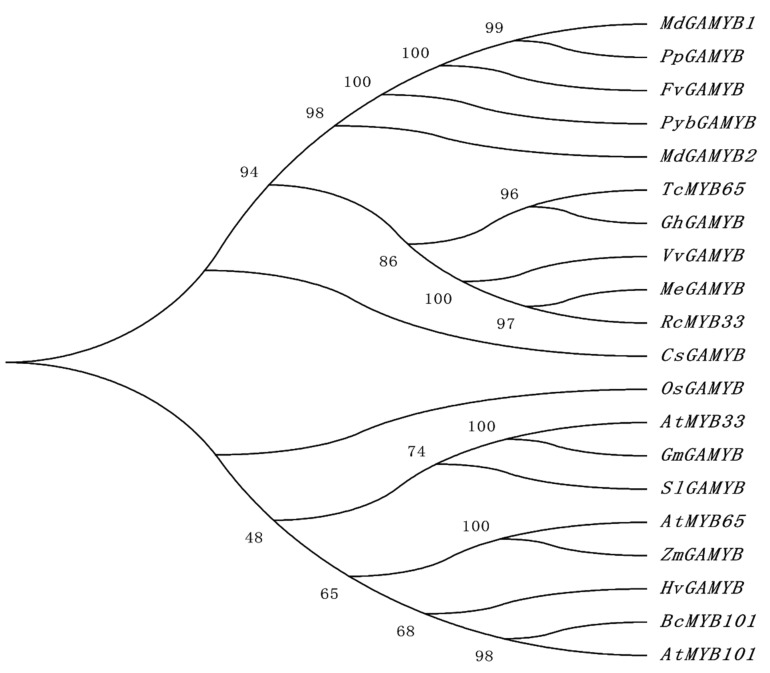
Phylogenetic analysis of GAMYB genes in diverse species. The phylogenetic relationship of GAMYB was constructed using MEGA7 software with 1000 bootstrap replications. In addition to the nine species used in multiple sequence alignment, *Malus domestica* (*Md*), *Fragaria vesca* (*Fv*), *Manihot esculenta* (*Me*), *Ricinus communis* (*Rc*), *Populus euphratica* (*Pe*), *Glycine max* (*Gm*), *Cucumis sativus* (*Cs*), *Pyrus* × *bretschneideri* (*Pyb*), and *Prunus persica* (*Pp*) were added to the construction of the phylogenetic tree.

To analyze the phylogenetic relationship of GAMYB and GAMYB-like proteins, an unrooted neighbor-joining (NJ) phylogenetic tree was constructed according to the full-length protein sequences in 17 species, consisting of three monocots *H. vulgare*, *O. sativa*, and *Z. mays*, and fourteen dicots *S. lycopersicum*, *A. thaliana*, *G. hirsutum*, *T. cacao*, *Malus domestica*, *Fragaria vesca*, *Manihot esculenta*, *Ricinus communis*, *Populus euphratica*, *Glycine max*, *Cucumis sativus*, *Pyrus* × *bretschneideri*, *Prunus persica*, and pak choi. As shown in the phylogenetic tree (Figure 2), the GAMYB proteins were divided into two larger groups; *OsGAMYB* and *CsGAMYB* formed separate branches, suggesting that GAMYB101 genes possibly exhibit functional differentiation in diverse species. Moreover, the result exhibited that GAMYB-like genes in *Arabidopsis*, soybean, tomato, corn, cereal, and pak choi may have originated from a common ancestral gene, with *BcMYB101* having the highest resemblance with *AtMYB101*.

### 2.3. Expression Patterns of BcMYB101 Gene

**Figure 3:**
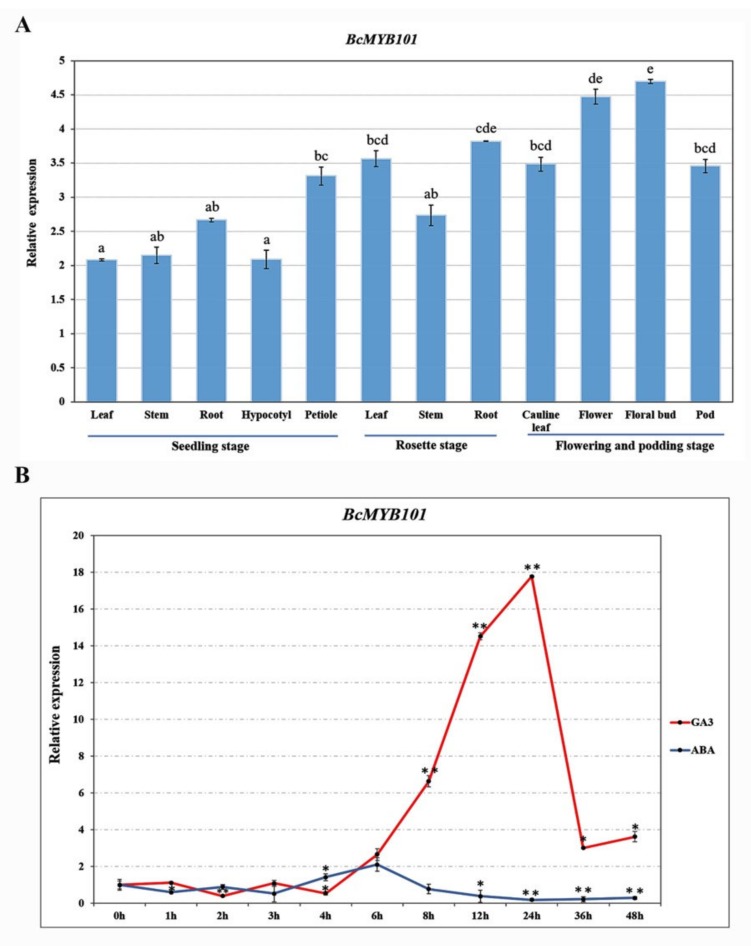
Expression profiles of *BcMYB101* in pak choi. (**A**) The transcript abundance of *BcMYB101* at seedling, rosette, flowering, and podding stages. ANOVA was selected for statistical analysis. Letters (a, b, c, d, e) represent the difference of expression level of *BcMYB101* gene in diverse tissues. (**B**) The expression analysis of *BcMYB101* under gibberellin (GA3) and abscisic acid (ABA) treatments. Sixth-leaf-stage pak choi seedling were exposed to GA3 (100 μM) and ABA (100 μM) treatments over a consecutive time course (0, 1, 2, 3, 4, 6, 8, 12, 24, 36, and 48 h). The vertical and horizontal axes represent the relative expression level and time, respectively. The transcript level of *BcMYB101* at 0 h was used as a control (expression value = 1). A *t*-test (two-tail) was selected for statistical analysis; * 0.01 < *p* < 0.05, ** *p* < 0.01.

MYB transcription factors extensively participate in the growth and development of roots, stems, leaves, and flowers. To verify the effects of the *BcMYB101* gene on pak choi development, its spatio-temporal expression was analyzed with qRT-PCR using total RNA from various tissues at different stages. As shown in Figure 3A, *BcMYB101* was widely expressed in different tissues, and a higher transcript level was identified in the petiole, leaf, root, and especially floral organs. Therefore, we speculated that *BcMYB101* may be mainly involved in the development of the leaf, petiole, root, and floral organs in pak choi. Additionally, the functional analysis of the *BcMYB101* gene promoter identified three conserved sequence (TAACAAR) present in the gibberellin response element (GARE) (Figure S2). Previous reports showed that the GARE plays a major role in mediating both ABA (abscisic acid) and GA regulation [33]. Therefore, we assumed that the *BcMYB101* gene responds to the phytohormones GA and ABA, and we studied the *BcMYB101* gene expression profile under 100 μM GA3 and ABA treatments using qRT-PCR. As we can see, the transcript levels of *BcMYB101* were significantly upregulated and downregulated in the time course of GA3 and ABA application, respectively, especially after 6 h. This result is consistent with GAMYB being a GA-regulated transcriptional factor (Figure 3B). 

### 2.4. Subcellular Localization of BcMYB101 Protein

**Figure 4:**
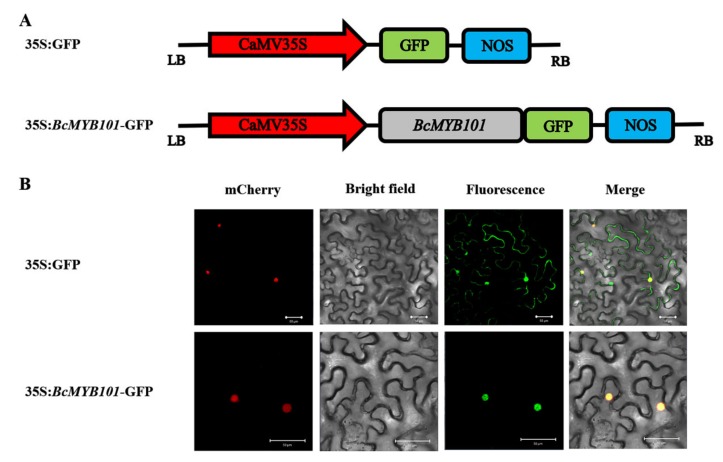
Subcellular localization of *BcMYB101*. (**A**) The construct of 35S:GFP (green fluorescent protein) and 35S:*BcMYB101*-GFP fusion protein. (**B**) The panels from left to right correspond to the mCherry (nuclear marker), bright-field, fluorescence, and merged fluorescence images of 35S:GFP and 35S:*BcMYB101*-GFP fusion protein. Scale bars = 50 µm.

To test whether *BcMYB101* is a nuclear protein, we constructed a fusion protein of 35S:*BcMYB101*-GFP for transient transformation via *Agrobacterium* injection methodology. The localization image of the *BcMYB101* protein was captured with a confocal laser scanning microscope at 48–72 h after *Agrobacterium* injection. As we can see, the fluorescence of 35S:GFP was displayed in the whole cell, while the 35S:*BcMYB101*-GFP protein was only enriched in the nucleus of tobacco epidermal cells, which indicated that *BcMYB101* is a nuclear-targeted protein with the potential characterization of a transcription factor (Figure 4).

### 2.5. Transgenic Arabidopsis with BcMYB101 Increased Leaf Number and Caused Downward-Curling Cauline Leaves 

**Figure 5:**
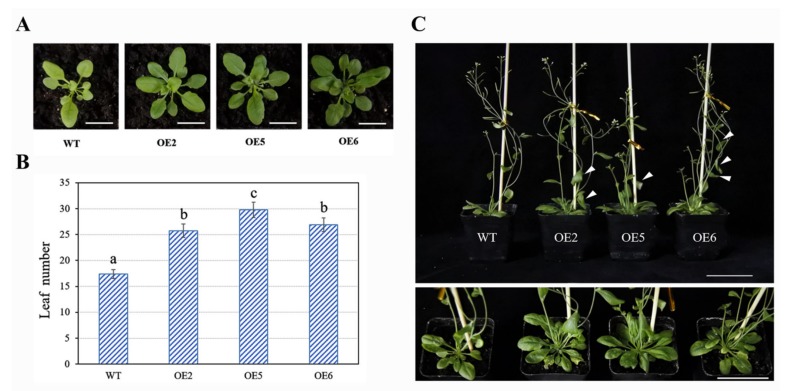
Phenotype of *BcMYB101* overexpression in *Arabidopsis*. (**A**) Leaf number difference of wild-type (WT) and transgenic plants overexpressing *BcMYB101*. Scale bar = 2 cm. (**B**) Rosette leaf number at bolting in the WT and *BcMYB101*-overexpressed lines OE2, OE5, and OE6. Error bars represent the standard deviation of the mean number of 20 plants for each line. ANOVA was used for statistical analysis. Letters (a, b, c) indicate the difference of leaf number in WT and *BcMYB101*-overexpressed plants. (**C**) Phenotype of downward-curling cauline leaves and higher leaf number in transgenic lines; the top view of the leaf is displayed at the bottom. Scale bar = 5 cm.

To investigate the function of *BcMYB101*, we firstly transformed *BcMYB101* into *Arabidopsis* to generate overexpression lines. Ten positive lines were identified by PCR amplification using specific primers and GUS (β-glucuronidase) staining, which were termed OE1 to OE10 (Figure S3A,B). We selected three T3 transgenic lines (OE2, OE5, OE6) with more prominent GUS staining for further research. Then, the expression levels of *BcMYB101* were identified using qRT-PCR, which showed that the transcript abundance of *BcMYB101* was significantly higher in transgenic plants than in the wild-type plant (Figure S3C). The rosette leaf numbers were increased until the time of bolting, and they were counted in overexpression lines #2, #5, and #6. As can be seen, the transgenic plants had significantly more leaves than control plants (Figure 5). An evident downward curling in cauline leaves was observed in the three lines (Figure 5C). These results indicated that *BcMYB101* might be involved in the regulation of leaf development.

### 2.6. Virus-Induced BcMYB101 Silencing Caused Upward-Curling Leaves in Pak Choi

**Figure 6:**
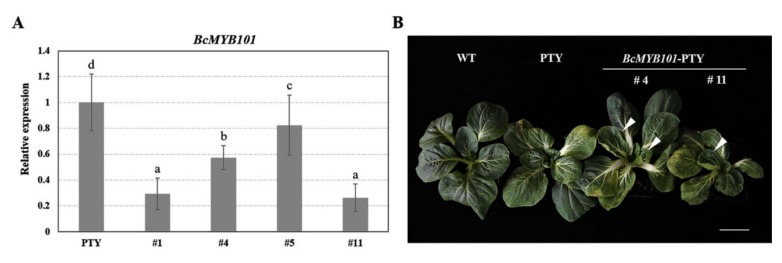
Virus-induced *BcMYB101* silencing in pak choi. (**A**) Expression levels of *BcMYB101* in gene silencing plants. Error bars represent the standard deviation of the mean number of three replicates. ANOVA was used for statistical analysis. Letters (a, b, c, d) represent the difference of expression level of *BcMYB101* gene in PTY and *BcMYB101*-silencing plants. (**B**) Upward-curling leaf phenotype in pak choi plants with *BcMYB101* silencing. Scale bar = 5 cm.

To furtherly verify the influence of *BcMYB101* on the leaf development of pak choi, loss-of-function plants were obtained using a virus-induced gene silencing (VIGS) assay. The phenotype of mosaic leaves is emblematic of a silencing plant, and this became increasingly evident in the positive plants of *BcMYB101*-PTY and PTY after two weeks. The potential loss-of-function plants were collected, and qRT-PCR (real-time PCR) was used for the assessment of the silencing efficiency of *BcMYB101*. As shown in Figure 6A, the transcript abundance of *BcMYB101* decreased by 70.7%, 42.8%, 17.6%, and 73.7% in *BcMYB101*-silencing plants #1, #4, #5, and #11, respectively, in comparison with the PTY plant. The phenotypic observations showed that *BcMYB101*-silencing plants exhibited distinct upward-curling leaves, especially the young leaves (Figure 6B). This result elucidated that *BcMYB101* has an important role in the formation of curly leaves.

### 2.7. Trans-Activation Activity Assays of BcMYB101

**Figure 7:**
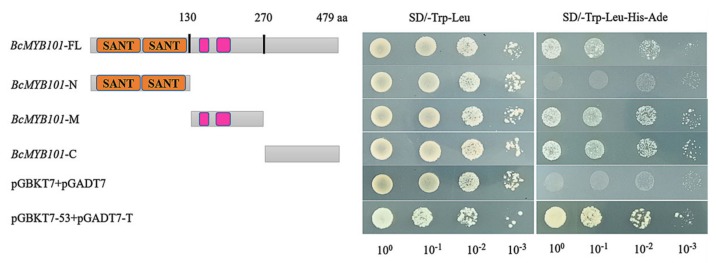
Trans-activation activity analysis of BcMYB101. Transcription activity assay of *BcMYB101* in yeast. *BcMYB101*-FL, *BcMYB101*-N, *BcMYB101*-M, and *BcMYB101*-C represent the full-length protein, as well as the regions encoding the N-terminal, middle, and C-terminal regions of *BcMYB101*, respectively, which were inserted into the pGBKT7 vector. The number of amino-acid residues is shown above the panel.

A previous study showed that 98.7% of OsMYB proteins and 98.47% of AtMYB proteins have transcript activation activity through gene ontology (GO) analysis [34]. In order to verify whether *BcMYB101* has transcript activation ability, trans-activation activity assays in yeast were carried out. The ORF of *BcMYB101* was inserted into the pGBKT7 vector (BD) containing the GAL4 DNA-binding domain, and the BD-*BcMYB101*-FL construct was generated. Then, the construct was co-transformed with an empty pGADT7 (AD) vector into yeast competent cells (Y2H Gold strain) and grown on selective solid medium. As expected, all yeast transformants grew on SD/-Trp-Leu medium. Furthermore, yeast transformants harboring BD-*BcMYB101* and AD were also able to grow on SD/-Trp-Leu-His-Ade medium, as well as the positive control (pGBKT7-53 + pGADT7-T), suggesting that *BcMYB101* has the capacity to activate reporter genes. On the other hand, the negative control (pGBKT7 + pGADT7) failed to activate the reporter genes (Figure 7). This result indicated that *BcMYB101* protein has transcriptional activation activity.

To further explore the transcriptional activation domain (TAD), the full-length *BcMYB101* was truncated into three fragments based on its structural features. As shown in Figure 7, only one fragment located in the N-terminal region failed to grow on SD/-Trp-Leu-His-Ade medium, indicating that *BcMYB101* has two transcriptional activation domains, which are located in the regions of *BcMYB101* from 131 to 270 amino acids, and 271 to 479 amino acids.

### 2.8. Screening and Validation of Interaction Proteins with BcMYB101 

**Figure 8:**
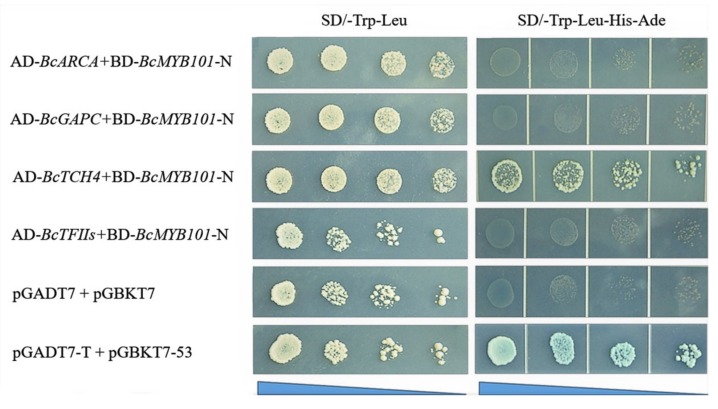
Verification of candidate proteins interacting with BcMYB101. pGADT7 + pGBKT7 and pGADT7-T + pGBKT7-53 were used as the negative and positive control. The blue triangle represents the concentration of yeast solution from high to low (10^0^–10^−3^).

To understand the regulatory network of *BcMYB101*, we firstly screened the potential interaction proteins using the cDNA library of pak choi. In this study, we obtained 30 positive clones, and four candidate genes (*BcARCA*, *BcGAPC*, *BcTCH4*, and *BcTFIIs*) were selected for Y2H verification according to functional annotation. In the Y2H assay, we discovered that only transformants containing the AD-*BcTCH4* plasmid could grow on both SD/-Trp-Leu and SD/-Trp-Leu-His-Ade plates among the candidate interaction proteins. The result indicated that *BcTCH4* can physically interact with *BcMYB101* to form a heterodimer (Figure 8). 

## 3. Discussion

The leaf plays important roles in the yield and quality of crops for its important function in photosynthesis. For higher plants, leaf development is one of the basic processes to ensure fine photoautotrophic growth, and this mechanism was established for coordinating the formation of leaf polarity [35]. The leaf morphology development of pak choi, as an important leafy vegetable, will significantly affect its yield and value. In this study, we identified a GAMYB gene associated with leaf morphology from Pak-choi, which we termed *BcMYB101* due to its protein structure having most similarity with *AtMYB101* in *Arabidopsis*. 

GAMYB transcription factors represent a subgroup of *Arabidopsis* MYB genes, which were firstly identified in barley [23,33,36]. GAMYBs act as critical promotors in the GA signal transduction pathway, and they play a crucial role in diverse biological process, including flower induction, anther development, seed germination, and stem elongation, regulated by miR159 [24,25,37,38,39]. In *Arabidopsis* and cereals, GAMYBs are required for GA-mediated programmed cell death in the tapetum during anther maturation and in aleurone tissues during seed germination [27,36,40,41]. In recent studies, three *Arabidopsis* GAMYB genes, *MYB97*, *MYB101*, and *MYB120*, were proven to be highly expressed mature pollen tubes and pollen grains, sharing overlapping functions in controlling proper pollen tube reception [26]. In general, *MYB101* does not accumulate in leaves, and our finding showed that *BcMYB101* transcripts had low expression in juvenile leaves, but relatively higher expression in mature leaves. *BcMYB101* was significantly expressed in the floral organs, which is consistent with its functions in anther development and flower induction (Figure 3). 

Gibberellin (GA), as an important phytohormone, has significant effects on the development of seeds and flowers, as well as stem elongation and leaf expansion [42,43]. GA regulates the expression of *miR159* through mediating ubiquitination of the DELLA protein, and it further regulates the cleavage of GAMYB [17,37,41,44,45]. In this study, we investigated the response of *BcMYB101* to GA and ABA through hormone treatment using qRT-PCR. The results exhibited that the expression level of BcMYB101 was increasingly upregulated by GA3 and downregulated by ABA. To further understand the mechanism of *BcMYB101* response to GA and ABA, different concentrations of GA3 (0/50/100 μM) and ABA (1/5 μM) were used for overexpression lines. When grown on 1/2 MS medium containing 50 μM GA3, the main roots of the *BcMYB101* overexpression transgenic line seedlings were slightly longer than that of the wild-type plants, and the transgenic lines had more lateral roots than WT (Figure S4). On the other hand, when grown on 1/2 MS containing 1 μM ABA, the degree of inhibition in terms of root length in transgenic lines was relatively low compared to wild type, with similar results under 5 μM ABA treatment. These results confirmed that *BcMYB101* responds to GA and ABA, and it participates in the growth and development of plants.

Several lines of evidence implicated that GAMYB is involved in the process of flower development and seed germination. To explore the function of *BcMYB101*, we firstly transformed *BcMYB101* into *Arabidopsis* for overexpression. Phenotype observation exhibited that the number of leaves in transgenic plants with BcMYB101 was increased, and the cauline leaves displayed a significant downward curl. Then, to further verify the role of *BcMYB101* in leaf development, virus-induced *BcMYB101* silencing assays were implement in pak choi. In positive *BcMYB101*-silencing plants, the phenotype of upward leaf curl was observed. These data demonstrated that *BcMYB101* is involved in the regulation of leaf shape development. 

In addition, we performed yeast trans-activation assays and revealed that *BcMYB101* likely acts as a transcription activator, which is in line with previous findings [26]. Furthermore, we selected four potential interaction proteins (*BcARCA/GAPC/TCH4/TFIIs*) with *BcMYB101* using yeast cDNA library screening and functional annotation (Table S2). Among them, only *BcTCH4* had an interaction relationship with *BcMYB101*, determined via yeast two-hybrid verification, which is involved in cell-wall biogenesis and response to auxin, brassinosteroid, cold, and heat. It is documented that BR is widely involved in the regulation of plant morphology; thus, we speculate that *BcMYB101* may participate in the signaling pathway of BR through an interaction with *TCH4* to regulate leaf shape development. Nevertheless, the specific functional mechanism remains to be further studied. 

## 4. Materials and Methods

### 4.1. Plant Materials

The plant seedlings of pak choi cultivars “*suzhouqing*” used in this study were grown in a phytotron with a 16-h/8-h light/dark cycle at 24 °C/18 °C. To have a better understanding of the expression of *BcMYB101* at various stages and in various organs, the leaves, stems, roots, hypocotyls, petioles, flower buds, flowers, and siliques at the seedling, rosette, flowering, and podding stages were collected from each sample with three replications. For different treatments, healthy seedlings with six leaves were selected and sprayed with exogenous phytohormone, consisting of GA (gibberellic acid, 100 μM) and ABA (abscisic acid, 100 μM). The samples were collected after 1 h, 2 h, 3 h, 4 h, 6 h, 8 h, 12 h, 24 h, 36 h, and 48 h (0 h was used as a control). 

*Arabidopsis thaliana* ecotype Colombia was used to transform the overexpression of *BcMYB101*, grown in a light growth incubator with long-day conditions (16-h/8-h light/dark cycle at 22 °C/18 °C, 75% humidity). 

The seeds of *Nicotiana benthamiana* were sown in a flowerpot containing a matrix, grown in a climatic chamber with a 16-h/8-h light/dark cycle at 24 °C/19 °C, 70% humidity. After two weeks, the seedlings were transformed into trays with 32 holes and used for the transient transformation assay. 

### 4.2. Isolation, Sequence Alignment, and Phylogenetic Analysis

The ORF (open reading frame) of *BcMYB101* was cloned from the complementary DNA (cDNA) library of “*suzhouqing*” tissues using special primers designed based on the full-length sequences from the *A. thaliana* database (TAIR10, http://arabidopsis.org/index.jsp) and the Chinese cabbage database (BRAD, http://brassicadb.org/brad/) according to a previous report [46]. The ORFs of *BcARCA*, *BcGAPC*, *BcTCH4*, and *BcTFIIs* were also isolated using the same method. The DNA sequence of *BcMYB101* promoter (1524 bp) was isolated from the DNA library. The gene-specific primers are listed in Table S1. The elements of the *BcMYB101* promoter were analyzed using the PLACE online software (http://bioinformatics.psb.ugent.be/webtools/plantcare/html/). ExPASy (https://web.expasy.org/protparam/) was used for the prediction of the physicochemical properties of *BcMYB101* protein. The secondary structure was predicted using PSIPRED 4.0 (http://bioinf.cs.ucl.ac.uk/psipred/). The conserved motif distribution was analyzed using the MEME Suite, and 10 motifs were identified (Figure 1B).

Multiple sequence alignment was conducted using DNAMAN software, and a phylogenetic tree was constructed by MEGA 7.0 using the neighbor-joining method with 1000 bootstrap replicates.

### 4.3. Transient Expression of 35S:BcMYB101-GFP Protein

To verify the subcellular localization of *BcMYB101*, the full fragment of cDNA was amplified from the pEASYBlunt vector and inserted into the entry vector pENTR/D-TOPO. Then, the *BcMYB101* was subcloned into the pEarlyGate103 vector after *Mlu*I enzyme digestion using the Gateway^®^ cloning system (Invitrogen, Carlsbad, CA, USA). The recombinant plasmid 35S:*BcMYB101*-GFP (green fluorescent protein) and empty vector plasmid 35S:GFP were transformed into *Agrobacterium tumefaciens* (GV3101 strain) and injected into the *Nicotiana benthamiana* foliar epidermis, respectively. Then, a red nuclear marker plasmid was used to confirm the location of the cell nucleus. Fluorescence was observed using a confocal laser scanning microscope (Zeiss, LSM 780, Jena, Germany) at 48–72 h after *Agrobacterium* injection. 

### 4.4. Generation of Transgenic Lines in Arabidopsis

To obtain transgenic plants, the full-length cDNA of *BcMYB101* was inserted into a binary vector pTCK303; then, the recombinant construct was transformed into *Agrobacterium tumefaciens* strain GV3101, and *Arabidopsis thaliana* transformation was performed using the floral dip method [47]. Seeds of transgenic T0/T1/T2 lines were screened and grown on Murashige and Skoog (MS) medium with 20 mg/L hygromycin for 15–20 days, and then the healthy seedlings were transplanted into a square flowerpot. Finally, T3 transgenic lines were used for further observation and verification. The leaf number of wild-type (WT) and transgenic plants with *BcMYB101* was counted at the bolting stage, and 20 plants of each line were analyzed. ANOVA (analysis of variance) was used for statistical analysis.

For GA3 and ABA treatment, the seeds of WT and T3 transgenic lines were sterilized and sown on 1/2 MS medium, kept at 4 °C for 24 h, and then transferred to an illumination incubator. To observe the phenotype under GA3 and ABA treatment, seedlings grown on 1/2 MS for five days were transferred to 1/2 MS medium without or with GA3 and ABA, and the seedling phenotypes six days after treatment were observed and photographed. This experiment was conducted three times, and four replicates were employed for each.

### 4.5. Virus-Induced Gene Silencing (VIGS)-Mediated Silencing of BcMYB101 in Pak Choi

To acquire the silencing plants, the virus-induced gene silencing assay (VIGS) was implemented according to a previous report [48]. The 80-nt *BcMYB101*-specific palindromic DNA fragment (5′–TTA ACA ATG ATG ACG AGT TTG AGT TTA ATT CAT TTC AAT TAA TTG AAA TGA ATT AAA CTC AAA CTC GTC ATC ATT GTT AA–3′) was primarily designed and synthesized by the GeneScript company (Nanjing, China). The sequence was inserted into the *SnaB*I site of the PTY vector which was linearized, and the *BcMYB101*-PTY construct was generated. Thereafter, PTY and *BcMYB101*-PTY plasmids were wrapped in gold particles and bombarded into two-week-old seedlings of pak choi using gene gun-mediated transformation (1300 psi, PDS-1000/He, Bio-Rad, Hercules, CA, USA). Four or five plants were bombarded for one plasmid, and three replicates were carried out. After two weeks, the virus symptoms on leaves became increasingly obvious with the initiation and expansion of new leaves, and this phenomenon was deemed the characterization for the initial screening of positive silencing plants. 

### 4.6. Trans-Activation Activity Assays

The transcriptional activation activity of BcMYB101 was studied using a yeast two-hybrid system. The full-length cDNA of *BcMYB101* was fused to the 3′ end of the GAL4 DNA-binding domain of the pGBKT7 (BD) vector, and BD-BcMYB101-FL was generated. The pGBKT7 (empty) vector and BD-WRKY33 served as a negative and positive control, respectively. The BD-*BcMYB101*-FL construct was co-transformed with an empty pGADT7 vector (AD) into yeast strain Y2H Gold, and the concrete operation scheme of yeast transformation was operated according to the instruction manual (Clontech, Mountain View, CA, USA). 

Afterward, we incised the cDNA of *BcMYB101* into three segments (N-terminal, middle, and C-terminal regions) based on its structural characteristics on account of the full-length *BcMYB101* having transcriptional activation activity, and we designated these segments as *BcMYB101*-N, *BcMYB101*-M, and *BcMYB101*-C, respectively. Three fragments were amplified from the BD-*BcKAN2*-FL construct and subcloned into pGBKT7, and the transcriptional activation domains (TADs) were identified as above.

### 4.7. Yeast Two-Hybrid (Y2H) Assays

To obtain the potential interaction proteins, a cDNA library of pak choi was used for Y2H screening, and BD-*BcMYB101*-N acted as a bait protein. The BD-*BcMYB101*-N plasmid was co-transformed with the library plasmid into a yeast complement cell (Y2H Gold strain) and plotted on the SD/-Trp-Leu-His-Ade medium with X-Gal. The positive transformants were sequenced and annotated for further analysis. The detailed operations referred to the product manual of the Yeastmaker™ Yeast Transformation System 2.

We selected four candidate genes for further verification based on functional annotation (Table S2). For the Y2H assay, the CDSs (coding sequences) of candidate genes (*BcARCA*, *BcGAPC*, *BcTCH4*, *BcTFIIs*) were amplified from the pEASYBlunt simple vector and inserted into the AD vector. Then, the prey plasmids (AD-*BcARCA*/*BcGAPC*/*BcTCH4*/*BcTFIIs*) were co-transformed with the bait plasmid (BD-*BcMYB101*-N) into the Y2H Gold strain, and then plotted on both SD-Trp/-Leu and SD-Trp/-Leu/-His/-Ade medium. The primers in this assay are listed in Table S1.

### 4.8. qRT-PCR Analysis

The total RNA extraction, cDNA synthesis, and qRT-PCR procedure referred to a previous method [49]. A melting curve was used for examining the specificity of all reactions. The *Actin* genes of Pak-choi and *Arabidopsis* were used as internal control genes to normalize the transcript levels of *BcMYB101* in different tissues and transgenic plants [50]. The relative expression levels were calculated using the 2^−∆∆*C*t^ method [51]. Three biological and technical replicates were employed for each reaction. ANOVA (analysis of variance) and a *t*-test (two-tail) were used for statistical analysis. All primers used in this study are listed in Table S1.

## 5. Conclusions

In conclusion, we firstly cloned a GAMYB gene, *BcMYB101*, from Pak-choi, which have a higher expression levels in leaves, petiole, and floral tissues, and response to GA and ABA. Ectopic expression and virus-induced gene silencing assays demonstrated that *BcMYB101* participated in the regulation of leaf shape development. Furthermore, the yeast two-hybrid assays showed that BcMYB101 protein have trans-activation activity and one candidate protein, BcTCH4, was verified have an interacting relationship with BcMYB101 in yeast. These findings extend our understanding of *MYB101* in *Cruciferae* plants and provide a solid foundation for further research on the regulatory mechanism of leaf shape in *Brassica* or other species. 

## Supplementary Materials

Supplementary materials can be found at https://www.mdpi.com/1422-0067/21/8/2750/s1.
