# Supplementary material for: Cloning and Functional Analysis of BcMYB101 Gene Involved in Leaf Development in Pak Choi (Brassica rapa ssp. Chinensis)
Source: Int J Mol Sci. 2020 Apr 15;21(8):2750. doi: 10.3390/ijms21082750 (PMC7254494; doi:10.3390/ijms21082750)
Supplement: Supplementary file 1 [file ijms-21-02750-s001.zip › supplementary figure.docx]

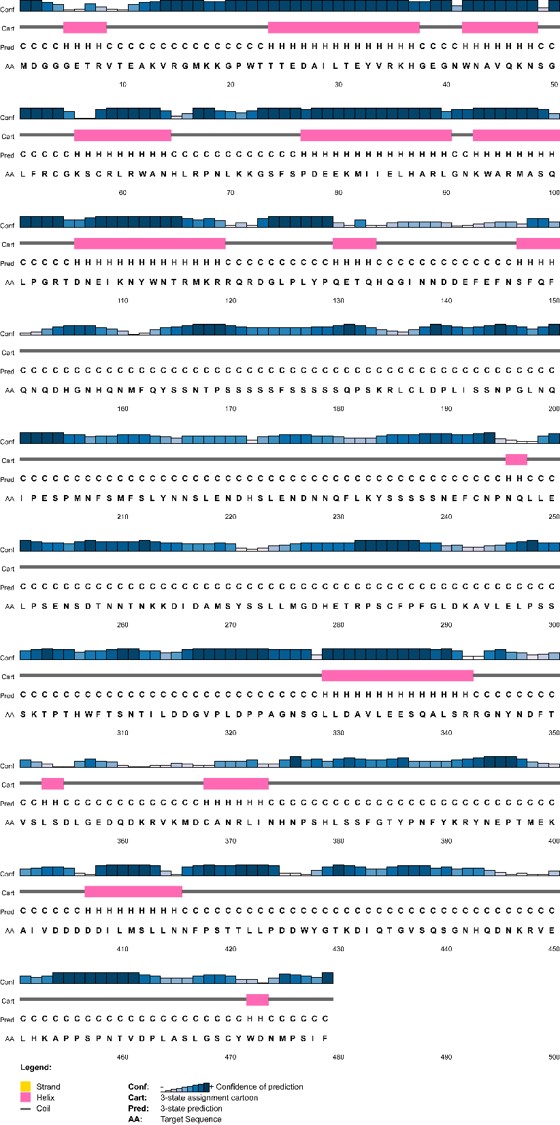


**Figure S1.** The secondary structure of *BcMYB101* protein.


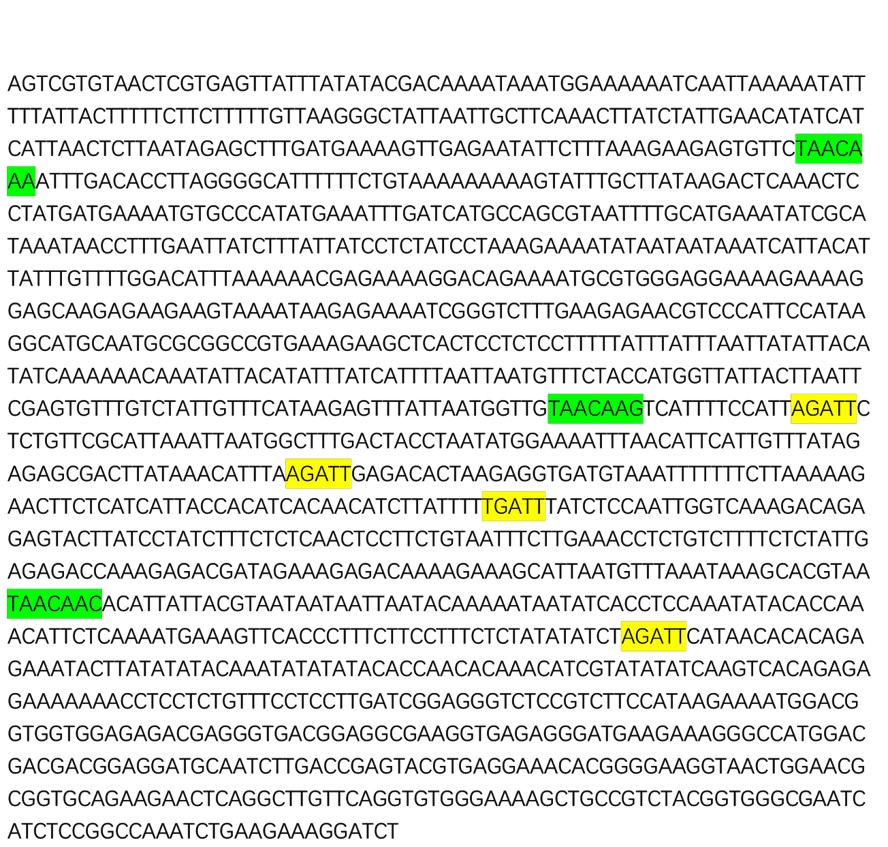


**Figure S2.** The GA-responsive element (GARE) and ARR1 elements position of *BcMYB101* promoter. The GARE and ARR1 element were marked with green and yellow fluorescence.


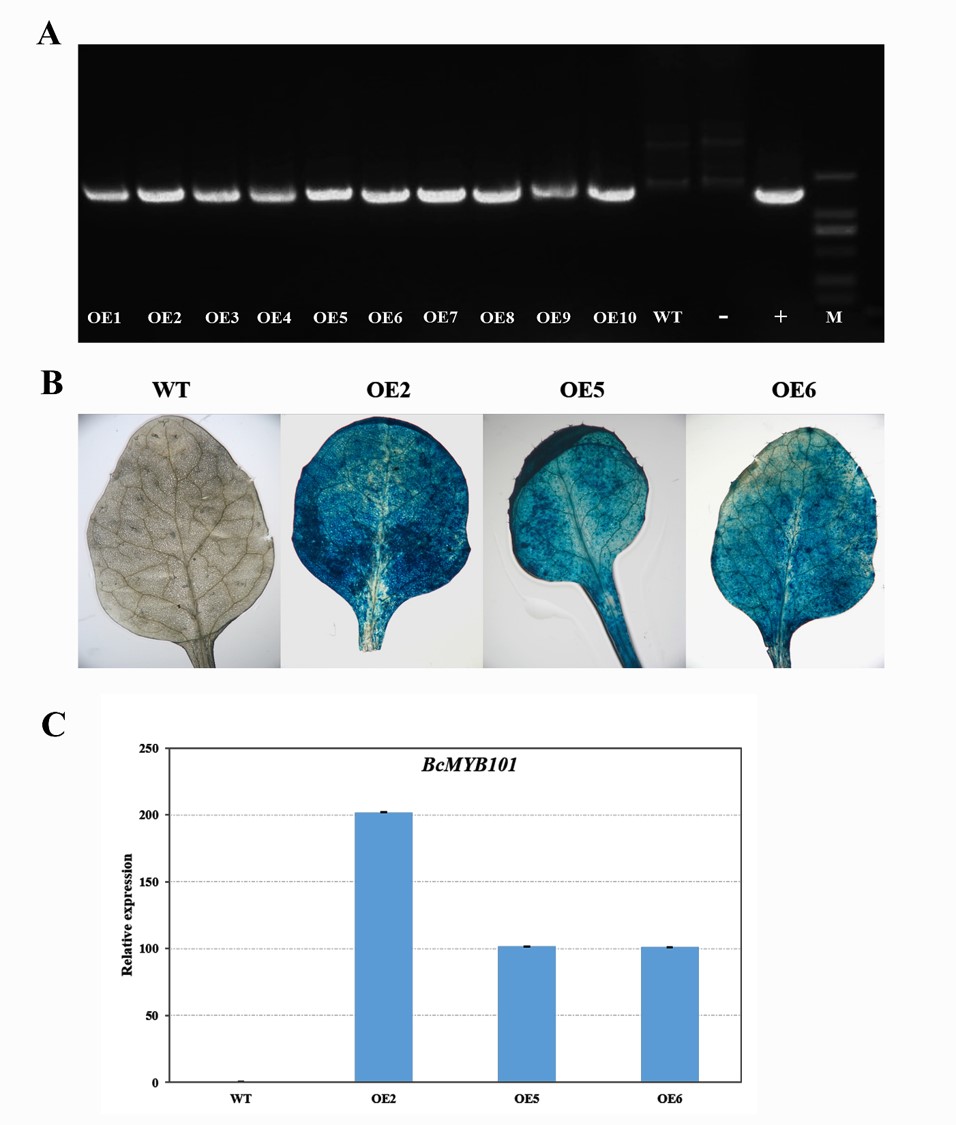


**Figure S3.** Identification of *BcMYB101* overexpression lines. The positive transgenic lines were identified using PCR amplification (**A**) and GUS staining (**B**), WT, +, - represents DNA templates of wild type *Arabidopsis*, plasmid of *BcMYB101*-pTCK303 construct, and ddH2O, respectively. M: DL2000 Marker. (**C**) Expression profile of *BcMYB101* in *Arabidopsis* overexpressed *BcMYB101*.


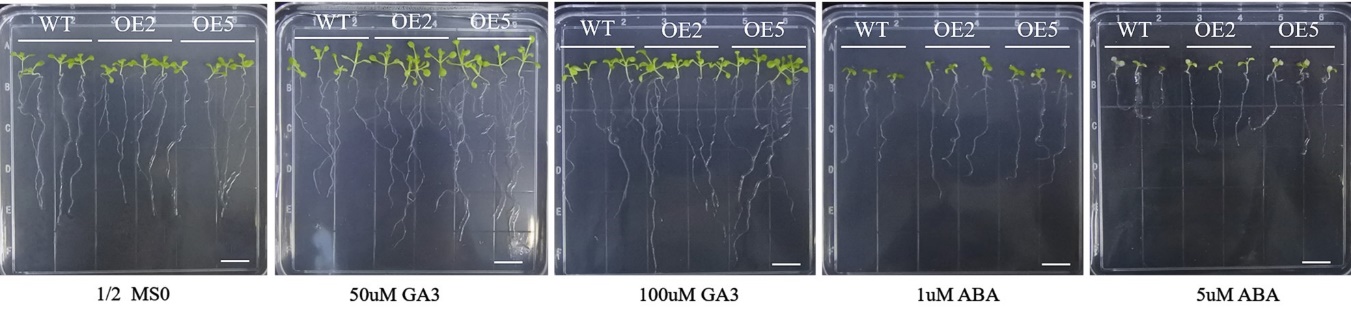


**Figure S4.** Phenotypic comparison. Seedlings grown on 1/2 MS medium with or without GA3 and ABA for 6 days.
